# Head–Body–Tail Segmentation of Poultry 3D Point Clouds Under Different Annotation Budgets: A Comparison of PCA-Based Rules and PointNet++-Based Methods

**DOI:** 10.3390/ani16162501

**Published:** 2026-08-11

**Authors:** Jianchao Yu, Hongyu Ding, Wentao Bi, Peiyi Lin, Xiaoze Yu, Tingting Jiang, Lizhe Ma, Haikun Zheng

**Affiliations:** 1 School of Mechanical and Energy Engineering, Guangdong Ocean University, Yangjiang 529500, Chinaedward.ding@gdou.edu.cn (H.D.); linpeiyi@gdou.edu.cn (P.L.); jiangtt@gdou.edu.cn (T.J.); malz@gdou.edu.cn (L.M.); 2 College of Engineering, Inner Mongolia Minzu University, Tongliao 028000, China; imunyxz@126.com

**Keywords:** poultry, 3D point cloud, head–body–tail segmentation, PCA rule, PointNet++, annotation budget

## Abstract

Head–body–tail segmentation of poultry 3D point clouds is an underexplored task in precision poultry phenotyping. This study constructs a segmentation-oriented evaluation setting to examine how supervision source and annotation budget affect the relative performance of a principal component analysis (PCA)-based geometric rule and PointNet++-based segmentation methods. The evaluation setting was derived from a previously published single-view poultry point-cloud dataset originally collected for body-weight prediction, to which point-wise anatomical annotations, PCA-derived pseudo-labels, fixed data partitions, and a 500-sample manually annotated test set were added for the present segmentation task. The results show that the PCA Rule achieved the best performance under pseudo-label supervision, indicating its value as a strong low-cost baseline when no human-annotated training labels are available. With increasing annotation budgets, learning-based methods progressively surpassed the rule-based baseline, while the performance gain became marginal beyond 1000 annotated samples. A cost-performance analysis further shows that PCA-based geometric partitioning provides a highly efficient solution in low annotation scenarios, whereas learning-based methods become more advantageous when moderate human supervision is available. As auxiliary application validation, the weight-regression experiments showed that anatomical decomposition did not outperform the complete whole-body representation under the present data and model settings. These findings provide empirical evidence on the relative behavior of geometric and learning-based segmentation methods under different annotation conditions and establish a reproducible evaluation basis for subsequent poultry point-cloud part segmentation studies.

## Simple Summary

Three-dimensional point clouds are digital collections of surface points that describe the body shape of live chickens. However, separating these data into head, body, and tail regions usually requires substantial manual labeling. This study compared a simple geometry-based rule with computer-learning methods under different amounts of manual annotation. The rule divided each chicken point cloud according to its main body direction and required no manually labeled training data. It performed best when no manual labels were available. As the number of manually labeled samples increased, the learning methods became more accurate, although the improvement became small beyond 1000 labeled samples. We also tested whether separating the head, body, and tail improved body-weight prediction. The complete whole-body point cloud remained more informative than the separated regions. These findings can help researchers choose a body-part separation method according to the available labeling budget, reduce unnecessary annotation work, and support the efficient development of automated poultry measurement systems.

## 1. Introduction

Poultry phenotyping is an important component of precision livestock farming, as it provides quantitative information for growth monitoring, health assessment, breeding selection, and production management [1,2,3]. In recent years, computer vision has been increasingly applied to poultry farming, enabling automated analysis of detection, posture, and behavior [1,2,4,5,6]. Compared with traditional manual observation, vision-based methods offer higher efficiency and better scalability in commercial farming environments.

Most existing poultry vision studies have concentrated on two-dimensional tasks, including object detection in challenging poultry environments [5], posture and pose estimation [7,8,9], behavior and lameness analysis [6,8,9], and local morphological measurement [10]. These studies have demonstrated the value of computer vision for poultry monitoring, but their outputs generally consist of bounding boxes, keypoints, behavioral categories, or a limited number of local measurements. They therefore do not provide point-wise anatomical partitions of the complete poultry body.

Three-dimensional point clouds offer explicit geometric information and have been used for livestock body measurement, segmentation, and phenotypic prediction [3,11,12,13]. In poultry research, however, point-cloud analysis remains comparatively limited. Our previous study showed that single-view whole-body point clouds could be used to predict live chicken weight [14], but it treated the bird as a single geometric object. Consequently, the use of poultry point clouds for point-wise anatomical segmentation, particularly the separation of the head, body, and tail, has not been adequately examined.

Head–body–tail segmentation provides a coarse but anatomically interpretable representation of poultry body structure. The body region contains most of the trunk geometry and is therefore closely related to overall body size and conformation, whereas the head and tail are peripheral structures with different shapes, orientations, and degrees of feather coverage. Separating these regions can reduce the influence of peripheral structures when trunk-related geometric traits are analyzed and can also support the extraction of region-specific morphological features. Such a representation may provide a useful intermediate step between whole-bird detection and more detailed phenotypic analysis. However, its practical value should be evaluated for specific downstream tasks rather than assumed from anatomical interpretability alone.

This gap is not limited to the absence of a segmentation model. Point-wise manual annotation of three-dimensional data is labor-intensive [15], whereas geometry-based rules can be applied without annotated training samples. Existing studies do not establish how these two approaches compare when the amount and source of supervision are systematically varied. In particular, it remains unclear whether a deterministic geometric partition is sufficient when no manual training labels are available, how many manually annotated samples are required for a learning-based method to surpass such a rule, and whether performance continues to improve as the annotation budget increases.

A second unresolved question concerns the practical role of anatomical decomposition. Separating a poultry point cloud into anatomical regions may highlight region-specific geometric characteristics, but it may also remove global relationships among the regions. Therefore, anatomical decomposition cannot be assumed to improve downstream phenotypic prediction and requires empirical evaluation.

To address these questions, this study constructs a segmentation-oriented evaluation framework using a previously acquired single-view poultry point-cloud dataset. New point-wise head, body, and tail annotations are organized with fixed data partitions and a common manually annotated test set. A PCA-based geometric rule and several PointNet++-based variants are then compared under pseudo-label supervision and three levels of manual supervision. The purpose is not to propose a new segmentation backbone, but to provide a controlled assessment of how supervision quality and annotation quantity affect the relative performance of rule-based and learning-based approaches.

To examine the application-level consequences of anatomical decomposition, an auxiliary weight-regression experiment compares the complete whole-body representation with several part-aware alternatives. This experiment is not intended to introduce a new weight-prediction method or constitute a primary contribution. Part-specific encoding could potentially emphasize regional geometric variation that may be diluted in a complete whole-body representation, but it could also weaken prediction by removing spatial relationships among the anatomical regions. The experiment therefore evaluates whether separating the head, body, and tail preserves, complements, or weakens the global geometric information used for prediction.

The main contributions of this study are as follows:

✧ A segmentation-oriented evaluation setting is constructed for point-wise head, body, and tail segmentation of poultry 3D point clouds, incorporating newly organized human annotations, PCA-derived pseudo-labels, fixed data partitions, and a common manually annotated test set.
✧ A controlled comparison of a PCA-based geometric rule and PointNet++-based methods is conducted under different supervision sources and annotation budgets, revealing the transition from rule-based effectiveness without manual training labels to learning-based superiority under sufficient human supervision.
✧ As auxiliary application validation, a downstream experiment examines the information retained or lost when complete whole-body geometry is replaced or supplemented by independently encoded anatomical regions.

### Related Work

Computer vision has been widely applied in poultry production for detection and monitoring tasks. Existing studies have shown that deep learning methods can effectively improve poultry detection performance under varying lighting, viewing angles, bird densities, and occlusion conditions [5]. In addition, machine-learning frameworks have been developed to detect, track, and classify individual behaviors in group-housed broilers [6]. These studies confirm the practical value of vision-based poultry analysis, but most of them are designed for 2D detection rather than fine-grained anatomical part segmentation.

Another major research direction concerns poultry pose estimation and body structure analysis. Deep neural network methods have been used for broiler pose estimation and behavior analysis [6,8,9], while multi-part detection has further extended poultry pose estimation to multiple anatomical landmarks [7]. Related studies have also explored lameness recognition from pose features [8,9]. These works demonstrate that poultry body structure can be effectively modeled by learning-based methods, but they mainly focus on keypoint localization, posture understanding, or behavior analysis rather than pointwise 3D segmentation.

Poultry phenotypic measurement has also attracted growing interest. Several studies have investigated local body-part measurement, such as shank length and circumference estimation [10], showing that anatomical information is highly relevant for poultry breeding and production management. However, these studies are typically designed for specific local traits, not for a unified semantic segmentation task covering the whole poultry body.

Compared with image-based studies, the use of 3D point clouds in poultry remains limited. Our previous work employed single-view point cloud information for live chicken weight prediction, demonstrating that poultry point clouds contain useful structural information for phenotypic analysis [14]. Independent research has also demonstrated the feasibility of estimating broiler weight from 3D depth images in commercial production settings [16]. More broadly, point cloud-based body measurement and segmentation methods have been explored in other livestock species, such as beef cattle and pigs [3,12,13,17]. These studies suggest that 3D geometric information is promising for animal body analysis [3,11,12,13]. Nevertheless, to the best of our knowledge, a standardized evaluation setting for head–body–tail segmentation in poultry 3D point clouds is still lacking. In particular, the relative performance of simple PCA-based geometric rules and PointNet++-based learning methods under different annotation budgets remains unclear. This gap motivates the present study.

Previous studies have shown that poultry body weight can be estimated from single-view whole-body point clouds, demonstrating the practical value of 3D geometric information for poultry phenotyping. However, it remains unclear whether anatomical decomposition can provide additional benefits for this task. Therefore, beyond benchmarking head–body–tail segmentation, this study further investigates how different anatomical regions contribute to downstream weight prediction.

## 2. Materials and Data

### 2.1. Data Source

The poultry point clouds analyzed in this study were obtained from the dataset reported in our previous work [14], in which the dataset was originally collected for live-chicken weight prediction. The source dataset comprised 2000 point-cloud records obtained through repeated measurements of 40 birds, including 20 Huainan partridge chickens and 20 Huxu chickens. In this manuscript, “bird” denotes a biological individual, whereas “point-cloud record” denotes one acquisition and is the unit used for data partitioning, model input, and record-level analysis. Data collection began when the birds were 40 days old and continued for 10 weeks. Measurements were conducted once per week, and each bird was captured five times at intervals during each session. Point clouds were acquired from a top-down view using an Intel RealSense D435 depth camera mounted above a 760 × 580 × 700 mm acquisition platform. The depth sensor had a field of view of 87° × 58°, and the average distance between the camera lens and the bird’s body surface was approximately 400 mm. Therefore, the 2000 records represent repeated measurements rather than 2000 independent birds. The source study did not report the sex composition, and sex was not used as a stratification variable in the present study. That dataset was originally collected to investigate live chicken body-weight prediction from single-view whole-body point clouds. No additional birds, imaging devices, or acquisition sessions were introduced specifically for the present study.

The current study uses the point clouds for a different research purpose. Whereas the previous work treated each chicken as a single geometric object for regression, the present study reformulates each point cloud as a point-wise semantic segmentation sample. Each point was assigned to one of three anatomical classes: head, body, or tail. PCA-derived pseudo-labels, human annotations, and fixed experimental partitions were then organized to support comparisons under different sources and quantities of supervision.

Accordingly, the data-related contribution of this study lies in task reformulation, point-wise anatomical annotation, and the construction of a controlled evaluation protocol rather than in new point-cloud acquisition. Details of the imaging equipment and original acquisition procedure are available in the original study [14], while the preprocessing, annotation, supervision, and evaluation procedures specific to the present segmentation task are described in the following sections.

### 2.2. Point Cloud Representation

Each input sample consists of a single-view 3D point cloud representing one individual chicken acquired from a top-down perspective. The dataset was organized so that each point-cloud file contained only one bird. Accordingly, the task investigated in this study is part-specific semantic segmentation within an individual-bird point cloud, in which every point is classified as head, body, or tail. It is not an instance-segmentation task for separating multiple birds within a shared scene. According to the original dataset description [14], the point cloud primarily captures the dorsal and upper-body surfaces of the chicken. Although the source files may contain additional point attributes, the core representation used in the present comparison is based on three-dimensional spatial coordinates.

**Figure 1:**
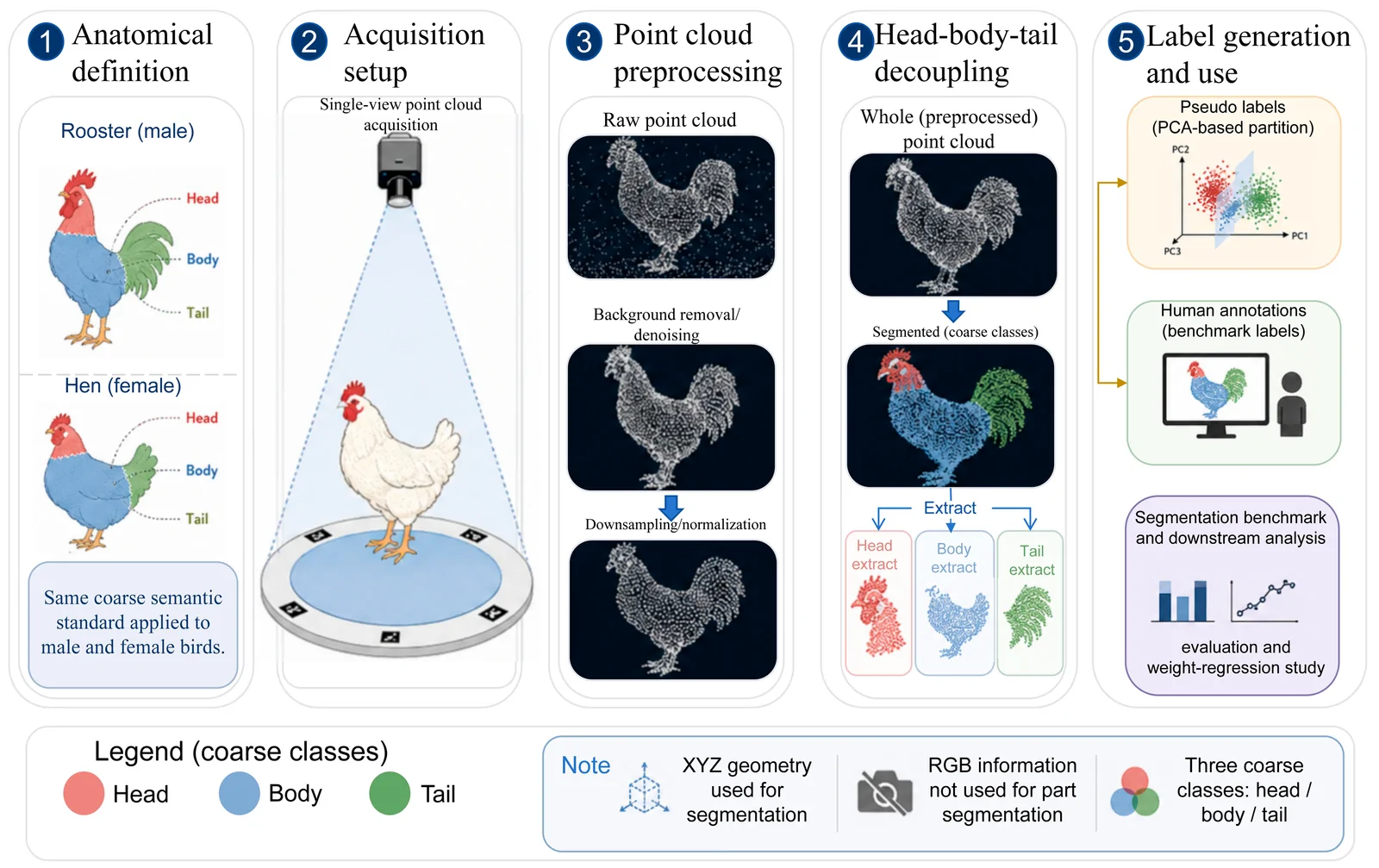
Experimental setup for poultry point cloud acquisition and examples of the preprocessing pipeline.

The original raw point clouds contained a large number of points and considerable noise. As reported previously [14], each raw point cloud contained approximately 400,000 points. A pass-through filter retained points within −0.30 to 0.30 m along the x-axis, −0.25 to 0.25 m along the y-axis, and 0.30 to 0.70 m along the z-axis, reducing each point cloud to approximately 15,000–25,000 points. Curvature-based downsampling was then performed by constructing a KD-tree, identifying the 20 nearest neighbors of each point, and estimating point normals using Open3D. Based on a mean normal-angle threshold of 30°, the points were divided into subsets with distinct and less-distinct geometric features. These subsets were uniformly downsampled using factors of 5 and 10, respectively, and subsequently merged, yielding approximately 4000 points while preserving the principal surface geometry. No pose normalization was applied. For the present experiments, each processed point-cloud record was standardized to 3072 points before model-specific input sampling. The experimental setup and the general preprocessing pipeline are illustrated in Figure 1.

For learning-based methods, the point clouds are further standardized to a fixed number of points during model input preparation. This standardization is necessary because neural network models such as PointNet++ require consistent input size across samples. In the benchmark, the point cloud data are therefore treated as unordered point sets, and a fixed number of points is sampled from each sample during training and evaluation. The basic PointNet++ baseline uses only the xyz coordinates as input, while the structurally enhanced variants may additionally incorporate rule-derived or geometry-derived auxiliary features.

### 2.3. Annotation Scheme for Head–Body–Tail Segmentation

To construct the segmentation benchmark, each point in a poultry point cloud was assigned to one of three anatomical regions: head, body, or tail. The three classes were encoded as pointwise labels, where 0 denotes the head, 1 denotes the body, and 2 denotes the tail.

Two types of labels were used in this study: pseudo-labels and human annotations. The pseudo-labels were generated automatically using a PCA-based geometric partitioning strategy, in which points were projected onto the principal axis and partitioned into head, body, and tail regions according to their relative positions. These labels were used for low annotation cost supervision experiments.

Human annotations were used for benchmark evaluation and full supervision experiments. Manual annotation was performed by partitioning each point cloud into the three anatomical regions and converting the segmented parts into pointwise labels. In this benchmark, pseudo-labels are used for low-cost training, whereas human annotations provide reliable evaluation and higher quality supervision.

### 2.4. Annotation Protocol

Manual annotation was performed using CloudCompare v2.13.2 (preview, 64-bit build). Each point cloud was rotated and inspected from multiple viewpoints rather than annotated only from a fixed top view. Polygonal selections, rather than three axis-aligned bounding boxes, were manually drawn to delineate the head, body, and tail regions according to the visible geometric transitions between adjacent regions. The selected points were subsequently exported and converted into point-wise class labels, where 0, 1, and 2 represented the head, body, and tail, respectively.

The objective of manual annotation was to obtain a consistent coarse semantic partition rather than to identify fine-grained anatomical landmarks. The same three-region definitions and annotation procedure were applied throughout the dataset. No sex-specific boundary rules were used because sex metadata were unavailable, and sex-stratified segmentation performance was therefore not evaluated. RGB information was not used as model input; all segmentation and weight-regression experiments used XYZ coordinates, with the enhanced segmentation variants using only additional geometry-derived features.

No records were manually excluded because of posture variation, partial occlusion, or imperfect geometric regularity. These difficult records were retained to preserve the variability observed under practical single-view acquisition conditions.

## 3. Experimental Methods and Design

### 3.1. Compared Segmentation Methods

**Figure 2:**
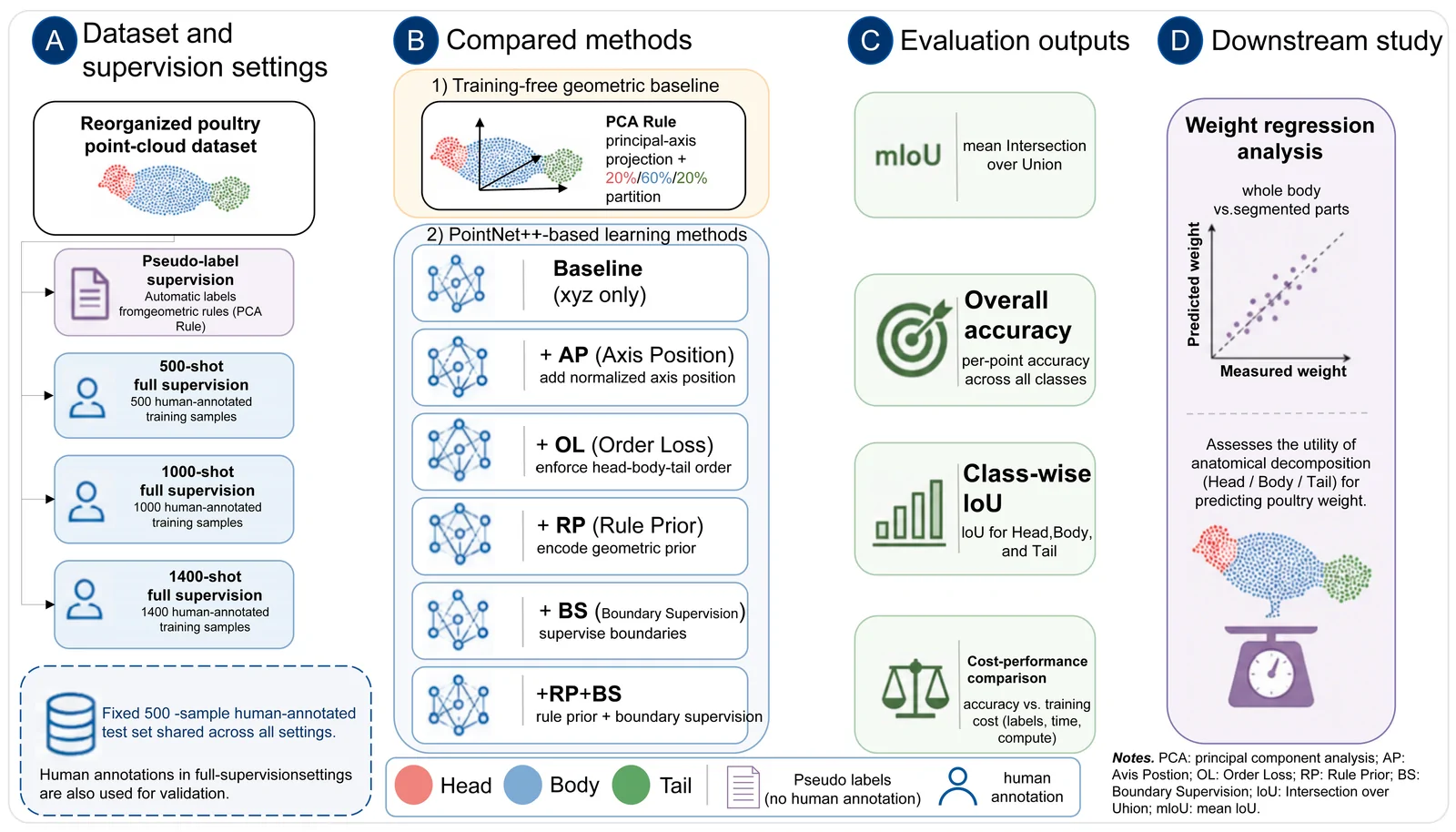
Comparison framework of PCA Rule-based and PointNet++-based methods for poultry head–body–tail segmentation.

To compare alternative approaches to head–body–tail segmentation of poultry 3D point clouds, we evaluated one geometry-based rule method and several PointNet++-based learning methods under consistent experimental settings. The compared methods include a PCA-based rule segmentation approach, a basic PointNet++ baseline, and several structurally enhanced variants. All learning-based methods aim to assign each point to one of three semantic regions, namely head, body, and tail. Figure 2 presents the PCA-based rule method and the PointNet++-based variants used in this study.

PCA Rule is a geometry-based segmentation method that does not require model training. Specifically, principal component analysis (PCA) is applied to each poultry point cloud to estimate its principal axis, which approximately corresponds to the head-to-tail direction. The points are then projected onto this axis and partitioned into head, body, and tail regions according to predefined positional ratios. This method serves as a strong, low-cost baseline in the benchmark.

PointNet++ Baseline is the basic learning-based segmentation model used for comparison [18]. It adopts the PointNet++ architecture and uses only the xyz coordinates of the input point cloud without any additional structural priors, auxiliary constraints, or boundary-aware mechanisms.

Baseline + AP augments the PointNet++ baseline with Axis-Position (AP) features. For each point, its normalized position along the PCA-derived principal axis is computed and appended to the input features. This variant is designed to provide the network with coarse global anatomical location information.

Baseline + OL introduces an additional Order Loss (OL) during training. This loss is intended to encourage the predicted head, body, and tail regions to follow a consistent anatomical order along the principal axis, thereby reducing anatomically implausible predictions.

Baseline + RP incorporates Rule-Prior (RP) features derived from the PCA Rule-based coarse partition. Instead of relying solely on raw xyz coordinates, the network is additionally provided with rule-guided prior information, enabling it to learn whether and how the coarse geometric rule should be refined.

Baseline + BS introduces Boundary Supervision (BS) to emphasize difficult transition regions, particularly the head-body and body-tail boundaries. This variant increases the training focus on boundary points so that the model can better distinguish adjacent anatomical regions.

Baseline + RP + BS combines both rule priors and Boundary Supervision. In this configuration, the network receives rule-guided structural cues while simultaneously being trained to pay greater attention to ambiguous boundary regions. This variant represents the most complete structurally enhanced learning-based method in the present benchmark.

### 3.2. Implementation Details

This section reports the parameter settings and computational procedures required to reproduce the compared methods; their conceptual differences have been described in Section 3.1.

For the PCA Rule-based partition, principal component analysis (PCA) was applied to the xyz coordinates of each poultry point cloud, and the first principal component was used as an estimate of the anatomical head-to-tail axis. Each point was projected onto this axis to obtain a one-dimensional anatomical coordinate. To make the coordinate comparable across samples, the projected values were normalized to a fixed interval after axis alignment. Following this normalized anatomical coordinate, the point cloud was partitioned into three consecutive regions using predefined positional ratios. Specifically, the first 20% of the projected range was assigned to the head region, the last 20% was assigned to the tail region, and the remaining middle 60% was assigned to the body region. In this way, the PCA Rule produced a deterministic coarse anatomical partition without any trainable parameters.

For the PCA Rule-based partition, PCA was applied to the XYZ coordinates of each point cloud, and the first principal component was used to estimate the dominant anatomical axis. Each point was projected onto this axis to obtain a one-dimensional coordinate. To resolve the sign ambiguity of the principal component deterministically, the point cloud was divided according to whether the initial projection was negative or non-negative. The axis was reversed when the mean z coordinate of the positive-projection subset was lower than that of the negative-projection subset. The aligned projection values were subsequently normalized to [−1, 1].

The normalized projection range was divided into three consecutive regions. The first 20% was assigned to the head, the central 60% to the body, and the final 20% to the tail. This symmetric definition reflects the operational assumption that the body occupies the largest central extent, whereas the head and tail form smaller terminal regions. The same fixed boundaries were applied to every record without record-specific adjustment. Therefore, the 20–60–20 partition should be interpreted as a reproducible operational baseline rather than as an anatomically optimal or universally applicable boundary.

To examine sensitivity to these thresholds without selecting parameters on the test set, the head and tail ratios were independently varied from 0.10 to 0.30 in increments of 0.05 on a validation set of 100 records, with the body ratio defined as the remaining proportion. The configuration with the highest pooled validation mIoU was locked before evaluation on the fixed external-test set. The prespecified 20–60–20 partition was retained for the primary benchmark because the pseudo-label and Rule-Prior experiments had been constructed using this common definition. The validation-selected configuration was therefore treated as a separate sensitivity analysis rather than as a replacement for the primary rule.

Because the sign of the principal axis is inherently ambiguous, an additional direction disambiguation step was applied before partitioning. The projected axis direction was aligned according to the geometric distribution of the point cloud so that the normalized coordinate followed a consistent anatomical order across samples. No manual exclusion was applied to difficult samples, and point clouds with posture variation, partial occlusion, or imperfect shape regularity were retained in the benchmark in order to preserve real-world variability.

All learning-based methods used a PointNet++ multi-scale grouping backbone. The baseline input consisted only of xyz coordinates, while the model-specific features and auxiliary objectives followed the definitions provided in Section 3.1. Before network input, each point cloud was normalized and randomly resampled to 2048 points during both training and evaluation to maintain a consistent input size.

All learning-based models were trained using Adam with an initial learning rate of 0.001, weight decay of 1 × 10^−4^, a batch size of 8, and cosine annealing to 1 × 10^−5^ over 60 epochs. The primary objective was unweighted point-wise cross-entropy. When anatomical-order regularization was enabled, a hinge-style ordering loss was added with a weight of 0.2 and a margin of 0.15. When Boundary Supervision was enabled, boundary points received a weight of 2.0, and an auxiliary two-class boundary loss was added with a coefficient of 0.2. NumPy and PyTorch random seeds were set to 42, and the checkpoint with the highest validation mIoU was retained for external-test evaluation. No explicit geometric augmentation, such as random rotation or scaling, was applied beyond random point sampling.

PointNet++ with multi-scale grouping was selected because its hierarchical neighborhood aggregation captures local geometric structures at multiple spatial scales, which is suitable for distinguishing adjacent anatomical regions [18]. The initial learning rate of 0.001 is consistent with the commonly used default setting of Adam [19]. The same batch size, epoch budget, and learning-rate schedule were applied to all PointNet++-based variants to reduce method-specific optimization bias. These settings are not claimed to be globally optimal.

The PointNet++ implementation used a multi-scale grouping encoder. The first abstraction layer sampled 512 centroids with radii of 0.1, 0.2, and 0.4 and neighborhood sizes of 16, 32, and 128. The second abstraction layer sampled 128 centroids with radii of 0.2, 0.4, and 0.8 and neighborhood sizes of 32, 64, and 128, followed by a global abstraction layer. The branch MLPs were [32, 32, 64]/[64, 64, 128]/[64, 96, 128] in the first abstraction layer and [64, 64, 128]/[128, 128, 256]/[128, 128, 256] in the second abstraction layer, with [256, 512, 1024] in the global layer. Feature propagation used [256, 256], [256, 128], and [128, 128, 128], followed by a 128-channel classifier, dropout of 0.3, and a three-class output layer.

To ensure consistent data organization across experiments, all benchmark settings used predefined train/validation/test splits stored as fixed split files. The random seed was fixed in the data splitting and model training procedures. Model selection for the learning-based methods was performed on the validation set, and the best checkpoint was retained for final evaluation on the fixed human-annotated test set.

To examine whether the conclusions depended on the use of a legacy backbone, the PointNet++ MSG baseline was additionally compared with two more recent point-cloud segmentation architectures, PointNeXt-S [20] and PointMLP [21], under the 1400-record supervision setting. The fixed development split comprised 1300 training records and 100 validation records, while 500 records were reserved for external testing. All models received XYZ coordinates only. During training and validation, 2048 points were sampled from each record, whereas all 3072 points were used for external-test evaluation. The models were trained for 60 epochs using Adam, an initial learning rate of 0.001, weight decay of 1 × 10^−4^, cosine learning-rate annealing, a batch size of 8, unweighted cross-entropy loss, and FP32 precision. The checkpoint with the highest validation mIoU was selected before external-test evaluation. Results were obtained using three independent seeds: 42, 2026, and 3407.

Unless otherwise specified, these implementation settings were held constant across the supervision conditions defined in Section 3.3.

### 3.3. Supervision Settings

To examine how the amount and source of supervision affected segmentation performance, four controlled supervision settings were constructed: one pseudo-label setting and three fully supervised settings using different numbers of human-annotated records. After reserving the fixed external-test set of 500 human-annotated records, 1500 records remained available for model development. The 500-, 1000-, and 1400-record settings represented approximately 33.3%, 66.7%, and 93.3% of this development pool, respectively. A validation set of 100 records was maintained in each fully supervised setting, resulting in training sets of 400, 900, and 1300 records. These settings represent low, intermediate, and near-complete annotation regimes while preserving the same validation and external-test protocols. They should be interpreted as controlled annotation-budget levels rather than universally optimal thresholds. The detailed data partitions are summarized in Table 1.

In the pseudo-label supervision setting, the learning-based models are trained using the full pseudo-labeled training set generated by the PCA-based coarse segmentation procedure. No human-annotated samples are used for training in this setting.

This setting should not be interpreted as unsupervised learning. The PointNet++-based models were trained with point-wise supervision, but the training labels were generated automatically by the PCA Rule rather than provided through human annotation. We therefore refer to this condition as pseudo-label supervision.

In the 500-shot full supervision setting, a total of 500 human-annotated samples are used for model development. Among them, 400 samples are used for training, and 100 samples are used for validation. The same fixed human-annotated test set is used for final evaluation.

In the 1000-shot full supervision setting, 1000 human-annotated samples are used for model development, including 900 training samples and 100 validation samples. Again, evaluation is performed on the same fixed human-annotated test set.

In the 1400-shot full supervision setting, 1400 human-annotated samples are used for model development, including 1300 training samples and 100 validation samples. The human-annotated test set remains unchanged across all settings in order to ensure fair comparison among different methods and annotation budgets.

The same 500 human-annotated point clouds and their corresponding pointwise ground-truth labels were used for testing in all four supervision settings. These test samples were kept separate from the training and validation data and were not used for model fitting, hyperparameter selection, or checkpoint selection. Therefore, differences in the reported test performance reflect differences among the segmentation methods and supervision settings rather than changes in the evaluation data.

The PCA Rule did not require a training or validation set. It was evaluated directly on the same fixed external-test set used for the learning-based methods and is therefore reported once as a training-free reference rather than as a separately trained method under each annotation budget.

### 3.4. Evaluation Metrics

The segmentation performance was evaluated using Intersection over Union (IoU), mean Intersection over Union (mIoU), and point-wise accuracy. Let Pc and Gc denote the predicted and ground-truth point sets for semantic class c, respectively, where c ∈ {head, body, tail}. The class-wise IoU was defined as:

$$IoU_{c}=\frac{\left|P_{c}\cap G_{c}\right|}{\left|P_{c}\cup G_{c}\right|}=\frac{TP_{c}}{TP_{c}+FP_{c}+FN_{c}}$$

where TPc, FPc, and FNc denote the numbers of true-positive, false-positive, and false-negative points for class c, respectively. The mIoU was calculated as the arithmetic mean of the class-wise IoU values:

$$mIoU=\frac{1}{C}\sum_{c=1}^{C}IoU_{c}$$

where C = 3 is the number of semantic classes. Point-wise accuracy was defined as the proportion of correctly classified points among all evaluated points:

Statistical analysis for the controlled architecture comparison was performed in Python 3.12.6 using NumPy 2.2.6 and SciPy 1.16.1. Pooled test-set mIoU from three independent training seeds (42, 2026, and 3407) was summarized as mean ± sample SD mean ± standard deviation across the three runs. Pairwise global mIoU differences were paired by seed and evaluated using exact two-sided sign-flip tests; *p* values were adjusted across the three model pairs using Holm’s method. To quantify uncertainty in record-level effects while reflecting both sources of variation, 95% percentile CIs for mean per-sample mIoU differences and mean per-record mIoU differences were obtained from 50,000 crossed bootstrap replicates that independently resampled the three training seeds and 500 sample records and 500 point-cloud records with replacement (bootstrap seed 42). As a secondary exploratory analysis, per-sample mIoU and per-record mIoU were averaged across seeds and compared using two-sided paired Wilcoxon signed-rank tests with Holm adjustment; matched-pairs rank-biserial correlation was reported as the effect size, with positive values favoring the first-named model. Bird identities were unavailable; therefore, sample-level intervals and tests record-level intervals and tests do not account for dependence among repeated records from the same bird and are not interpreted as bird-level inference. With only three independent training runs, the seed-level tests have limited power.

$$Accuracy=\frac{\sum_{c=1}^{C}TP_{c}}{N}$$

where N is the total number of evaluated points. Because the body class generally contains substantially more points than the head and tail classes, point-wise accuracy may be dominated by the majority class. Therefore, mIoU was used as the primary overall evaluation metric, while Head IoU and Tail IoU were reported separately to assess segmentation performance on the smaller and more anatomically challenging regions.

For the controlled architecture comparison, mIoU from three independent training seeds was summarized as mean ± standard deviation. Pairwise global mIoU differences were matched by seed and evaluated using exact two-sided sign-flip tests, with *p* values adjusted across the three model pairs using Holm’s method. To quantify uncertainty while reflecting both training-seed and record-level variation, 95% percentile confidence intervals for mean per-record mIoU differences were obtained from 50,000 crossed bootstrap replicates that independently resampled the three seeds and 500 point-cloud records with replacement. As a secondary exploratory analysis, per-record mIoU was averaged across seeds and compared using two-sided paired Wilcoxon signed-rank tests with Holm adjustment. Matched-pairs rank-biserial correlation was reported as the effect size. Because bird identities were unavailable, these record-level analyses do not account for dependence among repeated records from the same bird and should not be interpreted as bird-level inference.

Computational efficiency was evaluated using the mean per-sample segmentation time on the fixed test set. Timing excluded file loading but included the method-specific operations required to generate point-wise predictions from an in-memory point cloud. The PCA Rule was timed from principal-axis estimation to anatomical label assignment. PointNet++ inference was measured with a batch size of one after a warm-up stage. GPU synchronization was performed immediately before and after each timed PointNet++ inference to avoid underestimating runtime due to asynchronous CUDA execution. Mean and standard deviation were calculated across the test samples.

### 3.5. Annotation-Efficiency Analysis

Annotation efficiency was evaluated descriptively by comparing segmentation performance with the number of human-annotated samples used for model development. The PCA Rule and pseudo-label supervision settings were treated as requiring no human-annotated training samples, whereas the fully supervised settings used annotation budgets of 500, 1000, and 1400 samples. For each setting, the corresponding mIoU and point-wise accuracy were reported together with the annotation budget.

Model-training requirements were reported separately as a categorical methodological characteristic rather than being combined with annotation effort into a single cost score. No weighted composite cost index was used because manual annotation effort and computational training requirements have different units, and their relative importance depends on the available personnel, hardware, and deployment environment. Annotation efficiency was therefore interpreted from the observed performance improvement associated with increasing numbers of human-annotated samples.

### 3.6. Downstream Application Validation via Weight Regression

An auxiliary weight-regression experiment was conducted to evaluate the application-level consequences of anatomical decomposition. The experiment was not designed to establish a new weight-estimation benchmark or to assume that part-aware modeling would necessarily improve prediction accuracy. Instead, it examined whether anatomically separated regions could replace the whole-body representation, provide complementary information, or weaken prediction by disrupting global geometric relationships. Body-weight regression was selected because body weight is a continuous phenotypic trait available for each point-cloud record and had previously been modeled from the same data source using whole-body representations. This enabled a controlled comparison between whole-body and part-aware inputs while retaining segmentation evaluation as the primary focus of the study.

For the regression experiments, the input data were derived from the manually segmented poultry point-cloud dataset. Each sample consisted of three anatomically separated point clouds corresponding to the head, body, and tail regions. Human-annotated regions were deliberately used to isolate the effect of anatomical representation from errors introduced by an upstream automatic segmentation method. If PCA-derived regions had been used, the resulting regression performance would have reflected both segmentation error and the information value of anatomical decomposition, making these two effects difficult to distinguish. The body-weight label of each sample was obtained from the file name, following the same data source and label definition as in our previous whole-body point-cloud regression study. The downstream experiments therefore remained comparable to the previous whole-body regression setting in terms of data origin and evaluation protocol.

To maintain consistency with the previous study, the dataset was divided into training, development, and test subsets using a 60/20/20 split with the random seed fixed at 19. The same split protocol was applied to all regression methods. This setting ensured that the comparison among whole-body and part-aware regression strategies was performed under a unified data partition.

Seven regression strategies were considered in this study, and their main configurations are summarized in Table 2. First, a lightweight whole-body baseline was used as the direct regression reference from the complete poultry point cloud. Second, three lightweight part-aware fusion strategies were evaluated, including a softmax part-aware model, a fixed-weight part-aware model, and a Pure Concat Part-aware model. Third, three PointNet++-based strategies were tested, including the Whole-body PointNet++ model, the pure part-aware PointNet++ model, and the Hybrid Residual PointNet++ model. In the Hybrid Residual model, whole-body features were used as the main prediction branch, while head–body–tail features were introduced as an auxiliary residual correction branch. These model groups were selected to distinguish whether part-aware inputs improve prediction by replacing the whole-body representation or instead provide complementary information when introduced through an auxiliary residual branch.

To determine whether PCA-generated partitions could be propagated into the downstream task, a frozen-model input-substitution analysis was conducted on the fixed test subset of 393 records without retraining or model selection. For each record, the complete point cloud was reconstructed by merging the manually separated head, body, and tail files. Only XYZ coordinates were retained, and the reconstructed cloud was normalized once before applying the prespecified 20–60–20 PCA Rule. The PCA-defined head, body, and tail subsets were each sampled to 512 points using sampling seed 19 and supplied to the previously trained Pure Concat Part-aware and Hybrid Residual PointNet++ models. For the hybrid model, the whole-body input was held unchanged so that only the three local branches differed between the manual- and PCA-part conditions. Because the PointNet++ implementation used a random initial point in farthest-point sampling, the same FPS seed of 42 was reset for every test batch and comparison condition. This experiment was treated as a record-level sensitivity analysis of frozen models rather than as a new multi-seed training comparison.

The regression performance was evaluated using mean absolute error (MAE), mean absolute percentage error (MAPE), and Pearson correlation coefficient (Pearson r), following the evaluation protocol of the previous whole-body regression study. For the PointNet++-based models, training was performed with the Huber loss and cosine-annealing learning rate schedule. Since the multi-branch part-aware architectures are more computationally demanding than the previous single-branch whole-body model, the detailed training configuration was adapted to the current architecture while preserving the same evaluation metrics and overall regression objective.

These regression experiments were intended as auxiliary application validation rather than as the central benchmark contribution of this study.

## 4. Results and Analysis

### 4.1. Results Under Pseudo-Label Supervision

As summarized in Table 3, under pseudo-label supervision, the PCA Rule achieved the best overall performance, with an mIoU of 0.6874 and an accuracy of 0.8920, outperforming all PointNet++-based variants. Relative to the baseline, the PCA Rule improved mIoU by 0.0895 (15.0%). Among the learning-based methods, Baseline+RP+BS was the strongest variant, reaching an mIoU of 0.6165 and a Tail IoU of 0.4286, which were 0.0186 and 0.0520 higher than those of the baseline, respectively. In contrast, Baseline+AP+OL yielded only 0.5705 mIoU, lower than the baseline.

These results show that when no human-annotated training labels are available, direct PCA-based geometric partitioning remains the most effective solution. Although rule priors and Boundary Supervision improved the learning-based models to some extent, they did not surpass the rule-based baseline in this setting.

### 4.2. Results Under 500 Shot Full Supervision

With 500 human-annotated training samples, the learning-based models began to outperform the PCA Rule. The best result was obtained by Baseline+RP, which achieved an mIoU of 0.7250 and an accuracy of 0.9247, exceeding the PCA Rule by 0.0376 in mIoU (5.5%). The Head IoU also increased from 0.6530 to 0.7188. Compared with the baseline (0.6443 mIoU), adding Axis Position improved performance to 0.7107, while introducing Rule Prior further raised it to 0.7250. By comparison, Boundary Supervision alone reached 0.6749 mIoU.

These results indicate that 500 labeled samples are sufficient for learning-based methods to establish an advantage over the low-cost rule baseline. At this stage, Rule-Prior information is more beneficial than Boundary Supervision alone.

### 4.3. Results Under 1000 Shot Full Supervision

Further increasing the annotation budget to 1000 samples led to a substantial performance gain. Baseline+AP achieved the best result, with an mIoU of 0.7778 and an accuracy of 0.9375, improving on the PCA Rule by 0.0904 (13.2%). Compared with the best 500-shot result (0.7250), the best 1000-shot result increased by 0.0528. The improvement was particularly evident for the more difficult regions, with Head IoU reaching 0.7905 and Tail IoU reaching 0.6123.

At this annotation level, all learning-based variants outperformed the PCA Rule. This suggests that once a moderate amount of human supervision is available, data-driven methods can make more effective use of structural information than a fixed geometric partition.

### 4.4. Results Under 1400 Shot Full Supervision

Under 1400-shot full supervision, Baseline+AP+OL achieved the highest mIoU (0.7783), while Baseline+AP yielded the highest Head IoU (0.8081). Relative to the PCA Rule, the best model improved mIoU by 0.0909 (13.2%). However, the gain over the 1000-shot setting was marginal: the best mIoU increased from 0.7778 to 0.7783, a difference of only 0.0005.

This result suggests that the performance gain begins to saturate when the annotation budget approaches 1000 samples. Although additional annotations still maintain strong performance, their marginal contribution becomes much smaller than the improvement observed from 500 shots to 1000 shots.

A controlled comparison with more recent point-cloud segmentation architectures was also conducted under the 1400-record supervision setting. As summarized in Table 4, both PointNeXt-S and PointMLP achieved higher mean mIoU values than PointNet++ on the fixed external-test set. PointNeXt-S achieved the highest and most stable mIoU of 0.7656 ± 0.0035, followed by PointMLP at 0.7585 ± 0.0150, whereas PointNet++ obtained 0.7170 ± 0.0230. The corresponding absolute mIoU differences relative to PointNet++ were 0.0486 and 0.0415. PointNeXt-S also used fewer trainable parameters than PointNet++ and PointMLP. These results indicate that the evaluation setting can distinguish performance differences among architectures beyond the original PointNet++ family.

PointNeXt-S and PointMLP exceeded PointNet++ in all three matched runs. However, exact seed-level sign-flip tests were not significant after Holm correction (both adjusted *p* = 0.75), reflecting the limited statistical resolution of only three independent training runs. The crossed seed-by-record bootstrap estimated a mean per-record mIoU difference of 0.0340 (95% CI, 0.0137–0.0560) for PointNeXt-S versus PointNet++, and 0.0311 (95% CI, 0.0068–0.0613) for PointMLP versus PointNet++. PointNeXt-S and PointMLP did not differ significantly from each other. Because bird identities were unavailable, these record-level analyses should be interpreted as exploratory evidence rather than bird-level statistical inference.

Under the controlled 1400-record protocol, both recent architectures improved segmentation relative to PointNet++. PointNeXt-S achieved the highest and most stable mIoU (0.7656 ± 0.0035), followed by PointMLP (0.7585 ± 0.0150), compared with 0.7170 ± 0.0230 for PointNet++. The corresponding absolute improvements were 0.0486 and 0.0415. PointNeXt-S also required fewer parameters than PointNet++ and PointMLP. These results show that the evaluation setting can distinguish performance differences among more recent point-cloud architectures, although the comparison was conducted under a common protocol without architecture-specific hyperparameter optimization.

### 4.5. Comparison Across Annotation Budgets

**Figure 3:**
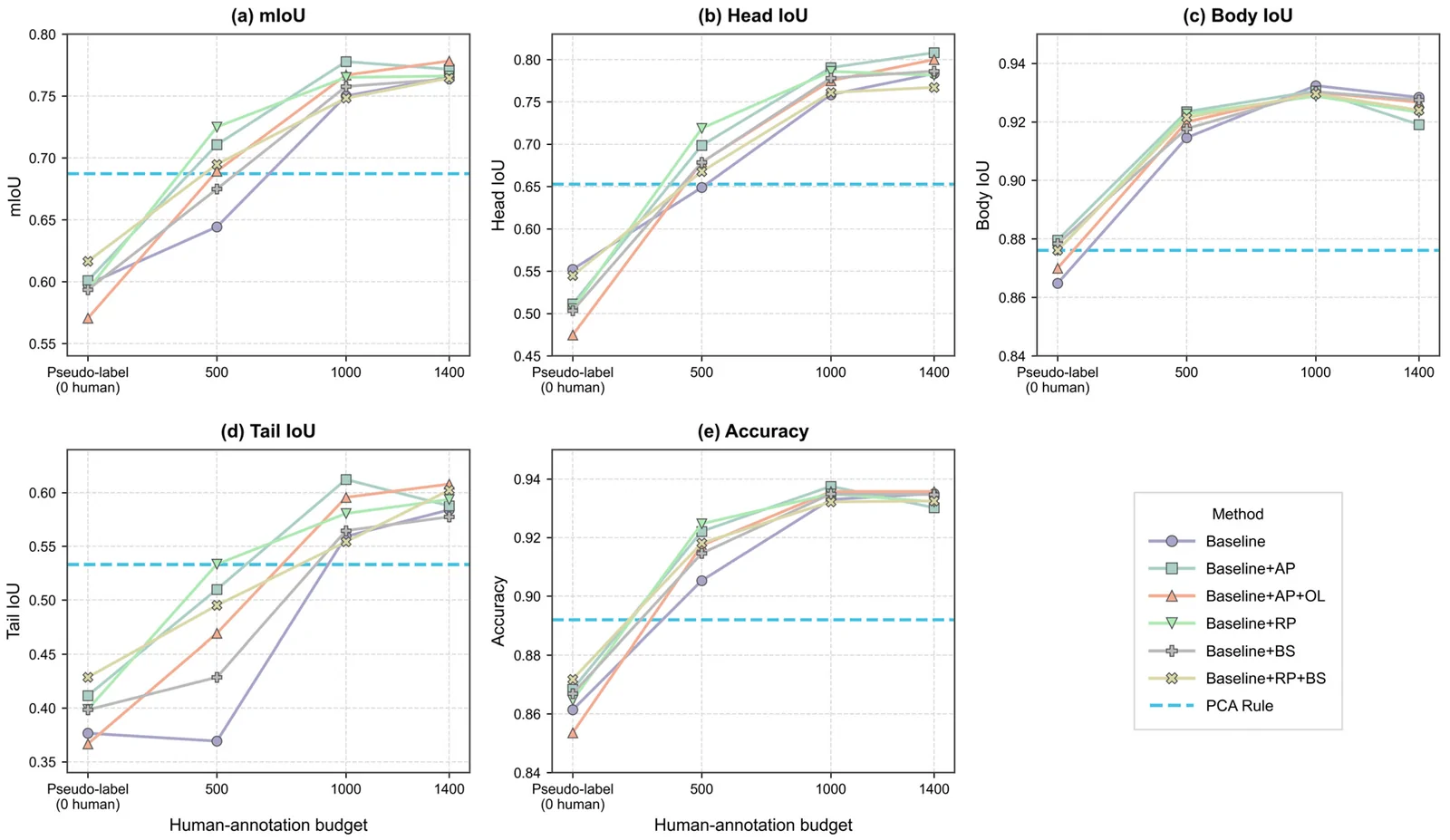
Segmentation performance across different supervision settings and human-annotation budgets. Panels (**a**–**e**) present mIoU, Head IoU, Body IoU, Tail IoU, and point-wise accuracy, respectively. Solid lines represent the PointNet++-based variants, whereas the dashed horizontal line represents the training-free PCA Rule. The pseudo-label condition uses no human-annotated training samples but employs labels generated automatically by the PCA Rule.

As illustrated in Figure 3, the relative performance of the compared methods varied substantially with the annotation budget. Under pseudo-label supervision, the PCA Rule remained the best-performing approach, achieving an mIoU of 0.6874, which was higher than all learning-based variants. Once human-annotated training data were introduced, the best learning-based method surpassed the PCA Rule at all three supervision levels. The best mIoU increased from 0.7250 at 500 shots to 0.7778 at 1000 shots, and then only marginally to 0.7783 at 1400 shots.

Figure 3 reorganizes the results according to the human-annotation budget rather than connecting different evaluation metrics on a common horizontal axis. Each panel tracks one metric across the pseudo-label, 500-shot, 1000-shot, and 1400-shot settings. The PCA Rule is shown as a constant reference because it does not depend on model training or annotation budget.

This trend indicates a clear transition from rule dominance to learning-based superiority as the annotation budget increases. At the same time, the gain becomes much smaller beyond 1000 annotated samples, suggesting that performance begins to saturate at this stage.

### 4.6. Annotation-Efficiency Analysis

**Figure 4:**
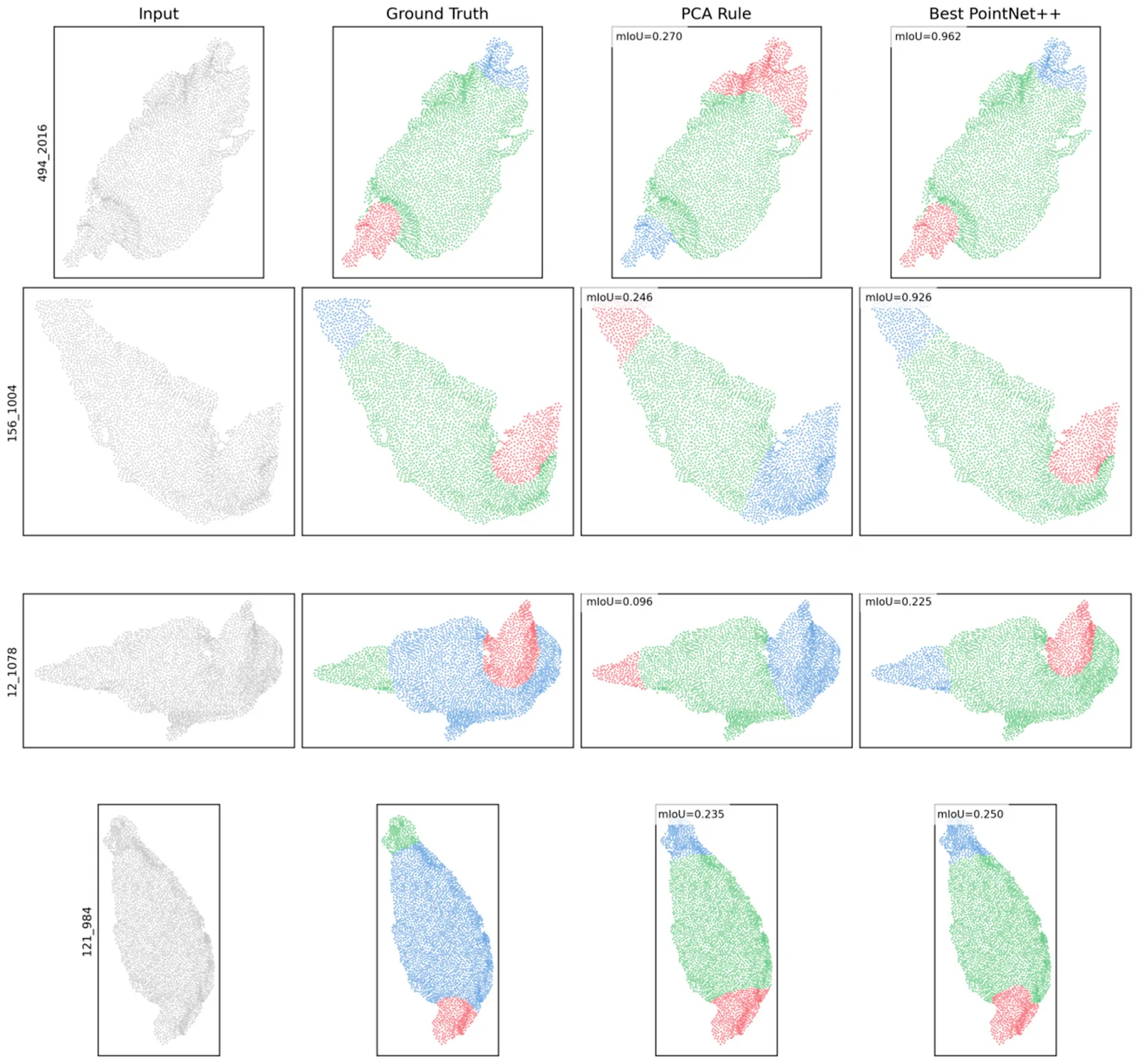
Qualitative examples of poultry head–body–tail segmentation results produced by the PCA Rule and the best learning-based model.

Representative qualitative comparisons between the PCA Rule and the best-performing learning-based model are shown in Figure 4. The examples illustrate the coarse but consistent anatomical partition produced by the PCA Rule and the more flexible boundaries produced by the model trained with human annotations. These visual results complement the quantitative comparisons reported in Table 3.

In the prediction columns, colors indicate the classes predicted by each method rather than the true anatomical identities. Consequently, incorrectly classified body regions may appear in the head or tail color in low-mIoU examples.

**Figure 5:**
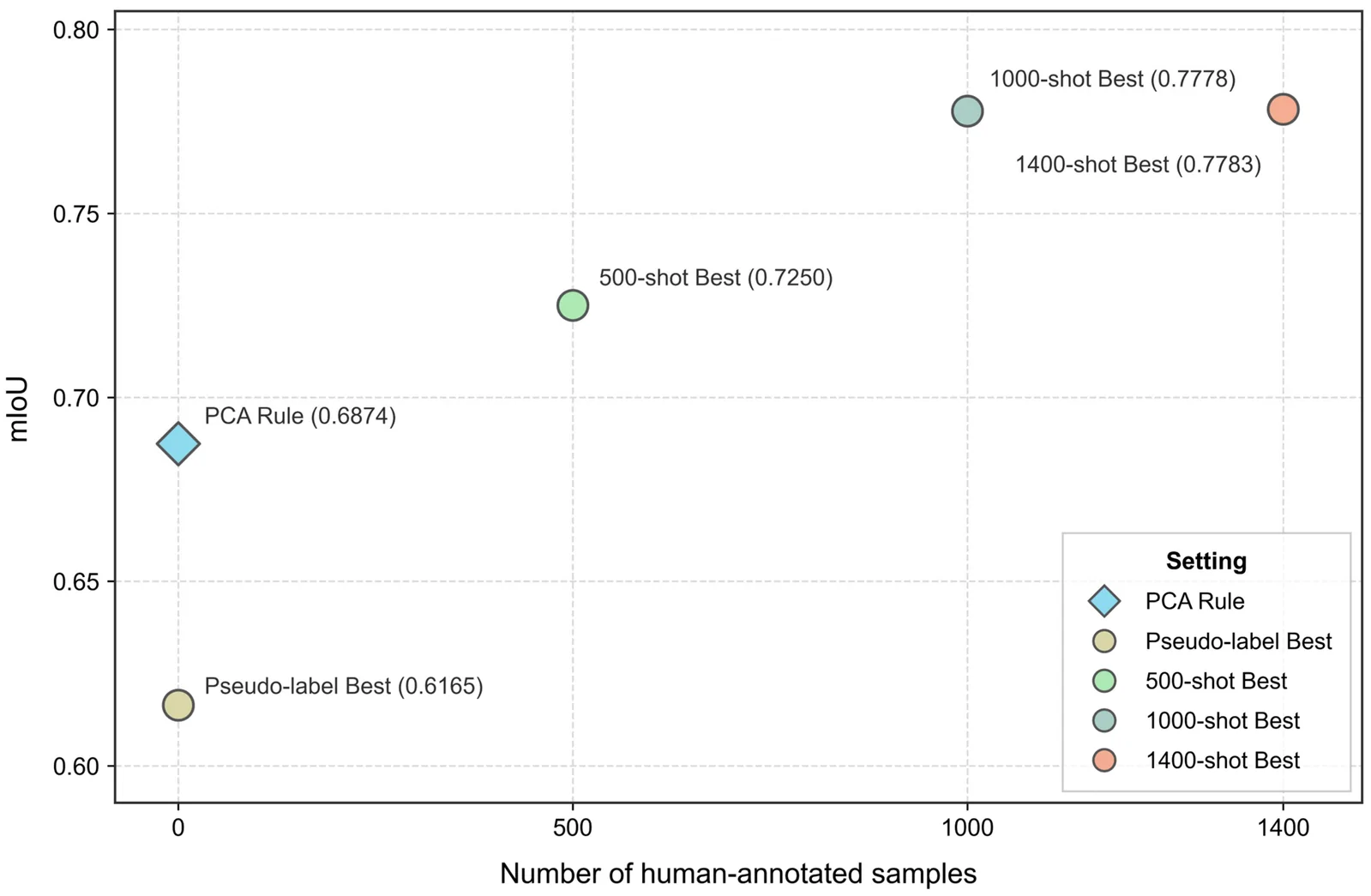
Relationship between human-annotation budget and segmentation performance under different supervision settings.

The relationship between annotation budget and segmentation performance is summarized in Table 3 and Figure 5. Without human-annotated training samples, the PCA Rule achieved an mIoU of 0.6874, whereas the best model trained entirely with PCA-derived pseudo-labels achieved an mIoU of 0.6165. When human annotations were introduced, the best mIoU increased to 0.7250 under the 500-shot setting and to 0.7778 under the 1000-shot setting. Figure 5 presents these results against the corresponding human-annotation budgets and distinguishes between methods that do and do not require model training.

Increasing the annotation budget from 1000 to 1400 samples improved the best mIoU only from 0.7778 to 0.7783. Thus, the additional 400 annotated samples produced an absolute mIoU increase of 0.0005 under the present dataset and model family. This result indicates diminishing marginal improvement beyond 1000 annotated samples in the current experiment. It should not, however, be interpreted as a universal annotation threshold because annotation efficiency may vary with dataset diversity, model architecture, and acquisition conditions.

To further compare the computational capacity required by each segmentation method, we profiled the PCA Rule and all PointNet++-based variants under the same 3072-point input setting used in the final manual test evaluation. For neural models, parameter count, FP32 model size, Conv/Linear FLOPs, median inference time, and peak CUDA memory were recorded. For the PCA Rule, which is a non-learning geometric procedure, the number of trainable parameters and neural network FLOPs were zero, and only the CPU rule partitioning time was measured.

Profiling was performed on a Windows 11 workstation equipped with a 13th Gen Intel(R) Core(TM) i7-13650HX CPU and an NVIDIA GeForce RTX 4060 Laptop GPU (8188 MiB; driver 566.07), using Python 3.12.6, PyTorch 2.5.1+cu121, CUDA 12.1, and cuDNN 9.1.0. All methods used a batch size of 1 and a 3072-point XYZ input. Randomly generated inputs of the required shape were used to isolate computational cost. After 30 warm-up runs, 100 repetitions were timed. Neural-network latency was measured with synchronized CUDA events under model.eval() and torch.inference_mode(); this timing covered the complete model forward call, including its sampling, neighborhood-query, and indexing operations, but excluded data loading, host-to-device transfer, and postprocessing. PCA latency was measured on CPU with time.perf_counter() and included normalization, PCA/SVD principal-axis projection, and the ratio-based partition. The reported value is the median per-sample and median per-record time; peak CUDA memory was obtained with torch.cuda.max_memory_allocated(). Conv/Linear FLOPs were estimated as twice the multiply-accumulate count for Conv and Linear layers and therefore do not include the non-Conv/Linear point-cloud operators. Because PCA and neural models were timed on different devices, these values are device-specific implementation measurements rather than a hardware-normalized speed comparison.

As shown in Table 5, the PCA Rule required no model storage or trainable parameters and completed one 3072-point partition in 0.76 ms on CPU. In contrast, the PointNet++-based models contained approximately 1.82 million parameters, required about 6.95 MB of FP32 model storage, and involved 8.55–8.61 GFLOPs per sample, with peak CUDA memory around 116 MB. These results indicate that the PCA Rule has a substantially lower computational requirement, whereas the learning-based models require additional GPU computation to obtain higher accuracy under human-annotated supervision.

### 4.7. Downstream Weight Regression Results

**Figure 6:**
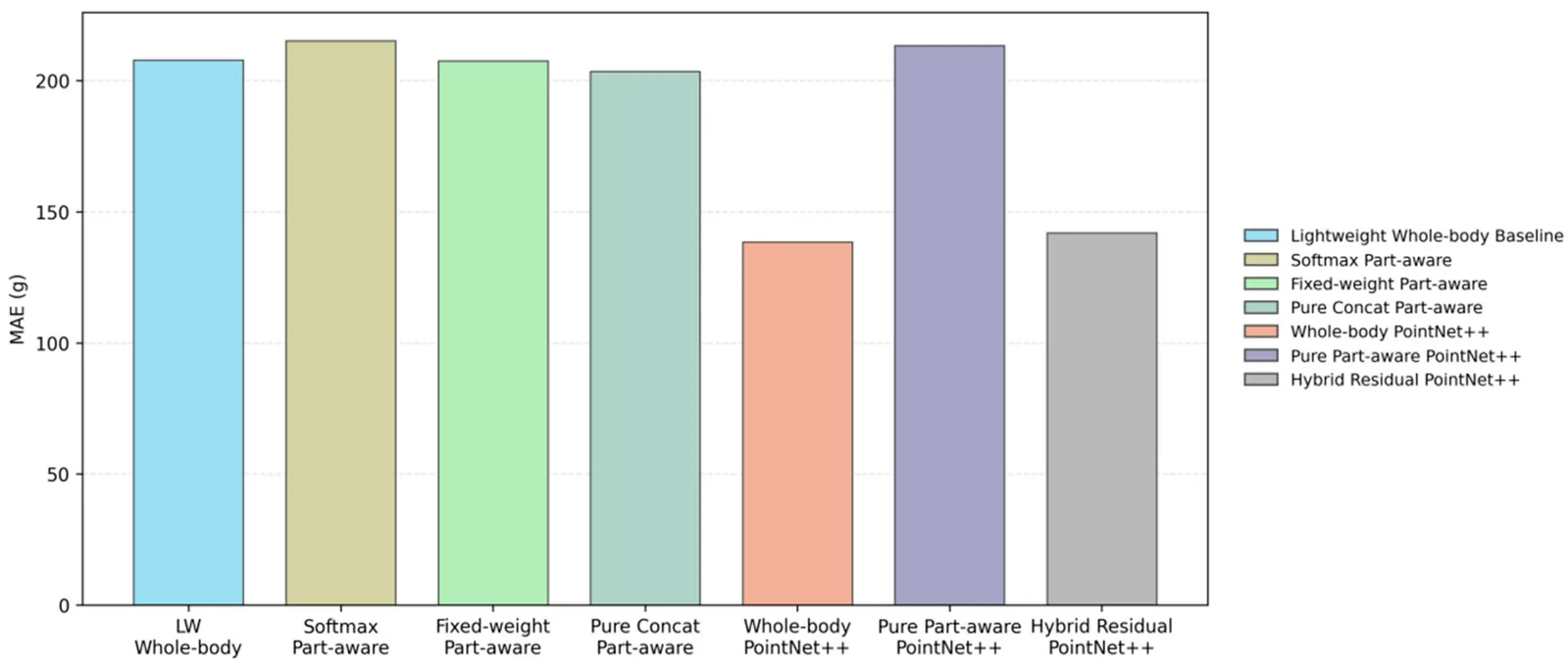
Comparison of downstream weight regression strategies in terms of MAE.

**Figure 7:**
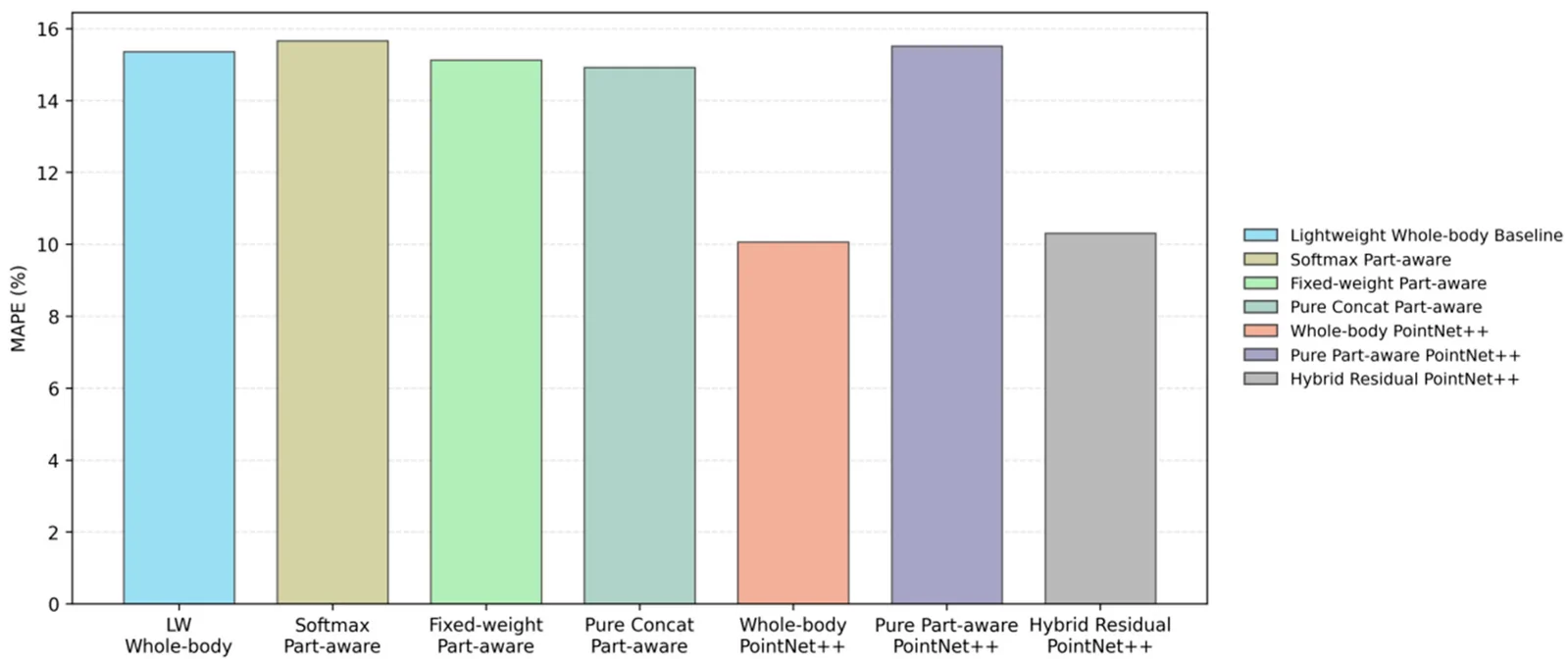
Comparison of downstream weight regression strategies in terms of MAPE.

**Figure 8:**
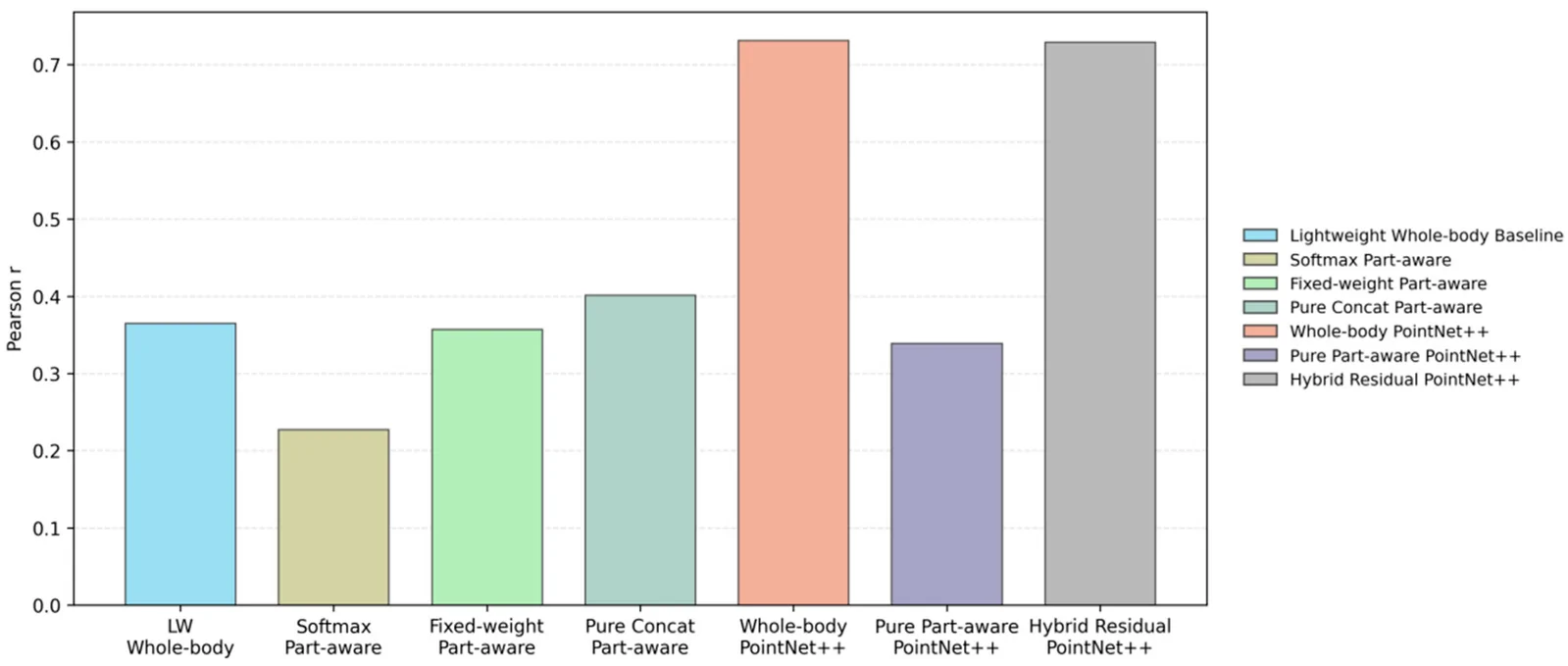
Comparison of downstream weight regression strategies in terms of Pearson correlation coefficient.

The results of the downstream weight regression experiments are summarized in Table 6. In the lightweight regression setting, the softmax part-aware model achieved the weakest performance, with an MAE of 215.31 g, a MAPE of 15.66%, and a Pearson r of 0.2275. Further inspection showed that the learned fusion weights rapidly collapsed to the body branch, indicating that the model tended to rely almost exclusively on body-related features. By contrast, the fixed-weight part-aware model improved the regression stability, but its performance remained close to that of the lightweight whole-body baseline. Among the lightweight part-aware strategies, the Pure Concat Part-aware model achieved the best result, with an MAE of 203.48 g, a MAPE of 14.91%, and a Pearson r of 0.4016, slightly outperforming the lightweight whole-body baseline. As shown in Figure 6, Figure 7 and Figure 8, the Pure Concat Part-aware model achieved the best performance among the lightweight part-aware strategies, whereas the Whole-body PointNet++ model remained the strongest overall regression model.

PointNeXt-S and PointMLP exceeded PointNet++ in all three matched runs. However, the exact seed-level sign-flip tests were not significant after Holm correction (both adjusted *p* = 0.75), reflecting the limited resolution of only three independent runs. The crossed seed-by-record bootstrap estimated mean per-record mIoU differences of 0.0340 (95% CI, 0.0137–0.0560) for PointNeXt-S versus PointNet++ and 0.0311 (95% CI, 0.0068–0.0613) for PointMLP versus PointNet++. PointNeXt-S and PointMLP did not differ significantly from each other. These findings provide evidence of improved record-level performance but should not be generalized as bird-level statistical inference.

**Figure 9:**
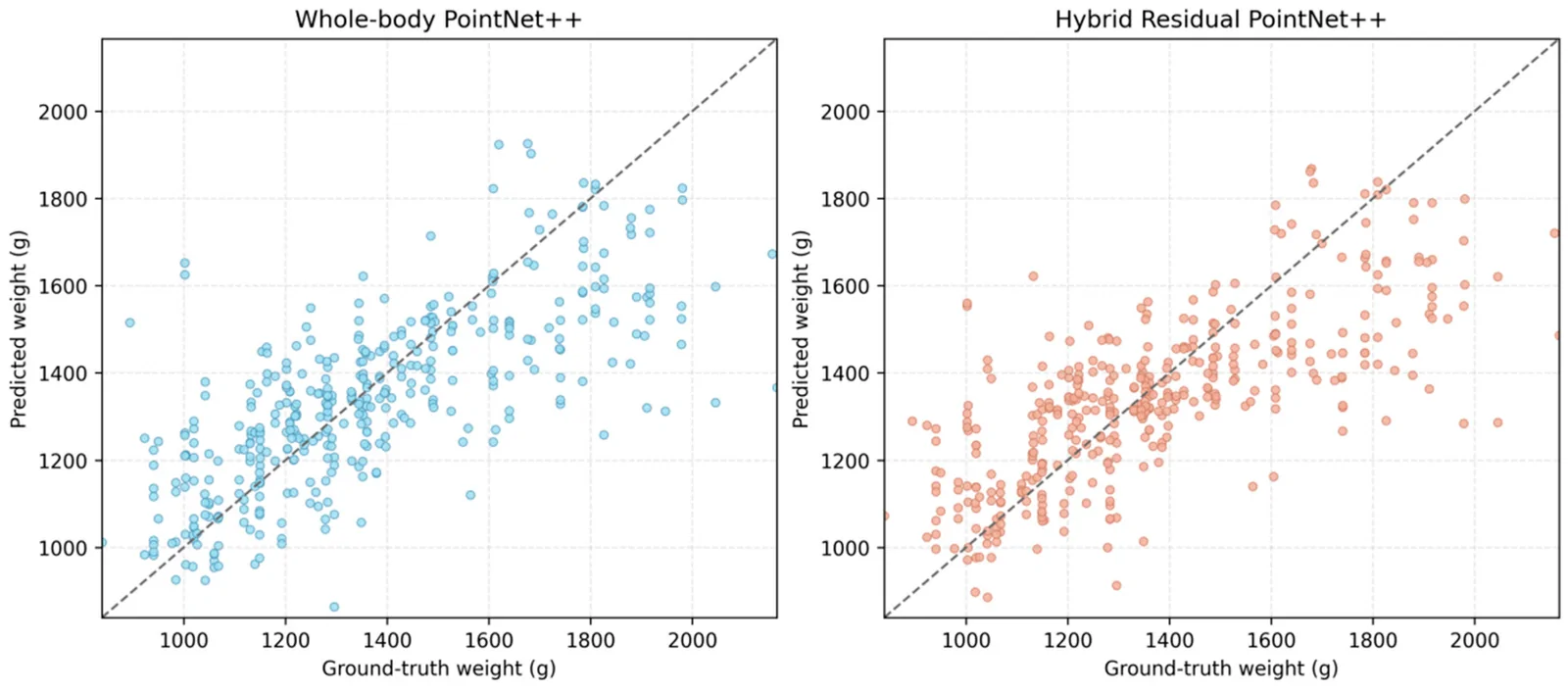
Predicted versus ground truth body weights for the Whole-body PointNet++ and Hybrid Residual PointNet++ models.

**Figure 10:**
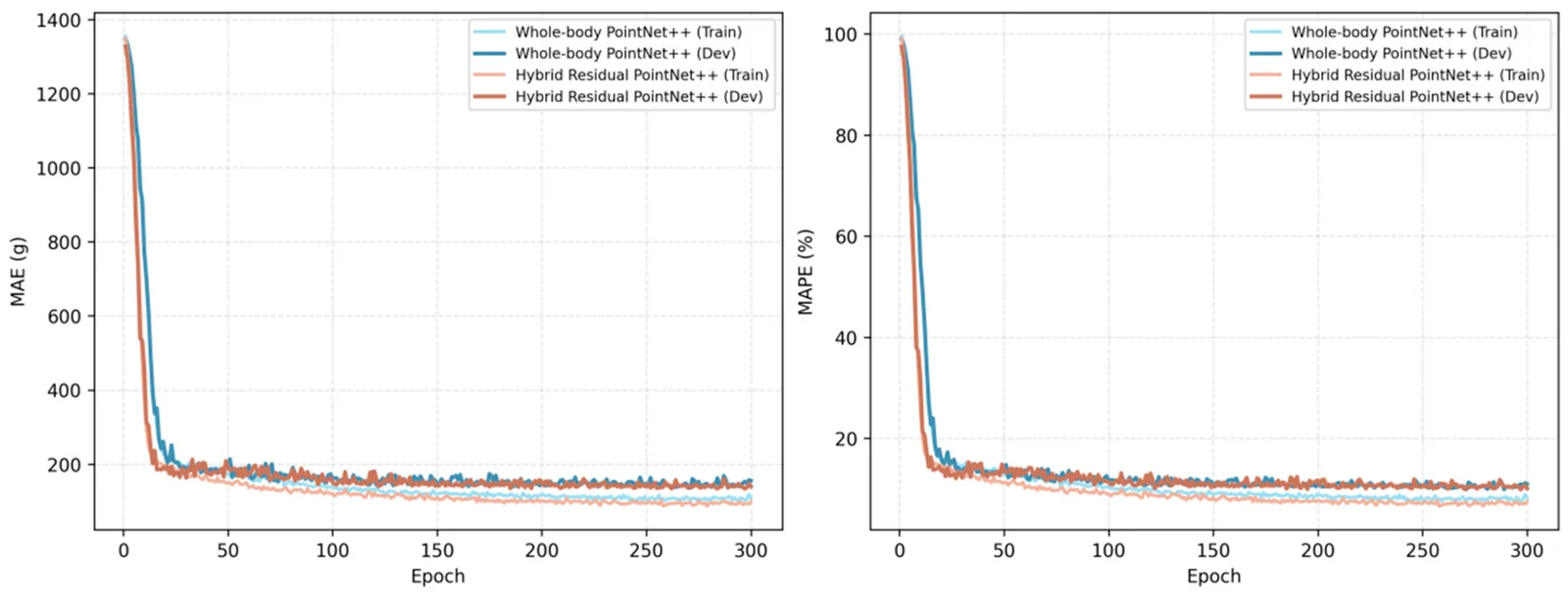
Training and development convergence curves of the Whole-body PointNet++ and Hybrid Residual PointNet++ models in terms of MAE and MAPE.

The PointNet++-based experiments revealed a different trend. The Whole-body PointNet++ model achieved the best overall performance, reaching an MAE of 138.56 g, a MAPE of 10.06%, and a Pearson r of 0.7316. In contrast, the pure part-aware PointNet++ model performed substantially worse, with an MAE of 213.43 g and a Pearson r of only 0.3390. This result suggests that directly replacing the whole-body representation with independently encoded part branches weakens the model’s ability to exploit global geometric information. The Hybrid Residual PointNet++ model partially alleviated this problem. By using the whole-body branch as the main predictor and the part-aware branch as an auxiliary residual correction, it achieved an MAE of 141.95 g, a MAPE of 10.31%, and a Pearson r of 0.7291, which was much better than the pure part-aware PointNet++ model and close to the Whole-body PointNet++ baseline. As shown in Figure 9, the Hybrid Residual PointNet++ model produced prediction patterns close to those of the Whole-body PointNet++ baseline, but did not exhibit a clear overall advantage. Figure 10 shows that the Hybrid Residual PointNet++ model followed a convergence trend close to that of the Whole-body PointNet++ baseline, although its final test performance did not surpass the whole-body model.

The frozen-model input-substitution analysis further compared the Hybrid Residual PointNet++ model using manually annotated and PCA-generated anatomical parts. As summarized in Table 7, the manual-part condition achieved an MAE of 142.79 g, a MAPE of 10.41%, an RMSE of 192.75 g, and a Pearson r of 0.7225. When the three local inputs were replaced with PCA-generated parts, the corresponding results were an MAE of 143.86 g, a MAPE of 10.50%, an RMSE of 192.78 g, and a Pearson r of 0.7224. The mean paired difference in absolute error, calculated as PCA minus manual, was 1.07 g, with a 95% bootstrap confidence interval from −2.03 to 4.17 g. The paired difference was not statistically detectable after Holm adjustment (*p* = 0.821). Thus, replacing the manually annotated local regions with PCA-generated regions produced a small numerical increase in prediction error but did not result in a statistically detectable change under the present frozen-model protocol. The manual-part result in this analysis differs slightly from the Hybrid Residual PointNet++ result reported in Table 6 because the substitution analysis reran inference with a fixed FPS seed to ensure identical stochastic sampling across the two input conditions.

Overall, the regression experiments suggest that body-related point cloud information provides the dominant predictive signal for poultry weight estimation, whereas head and tail regions contribute more weakly and mainly serve as complementary cues. More importantly, the results indicate that global whole-body geometry remains highly informative in single-view poultry weight regression. Therefore, under the current dataset and experimental setting, part-aware information appears to be more effective as an auxiliary refinement than as a direct substitute for the whole-body representation.

## 5. Discussion

### 5.1. Methodological Findings from the Segmentation Evaluation

This study does not propose a new point-cloud segmentation backbone. Instead, its contribution lies in constructing a controlled evaluation setting for poultry head–body–tail segmentation and in examining how rule-based and learning-based methods behave under different supervision sources and annotation budgets. By using fixed data partitions and a common manually annotated test set, the study enables direct comparison between a deterministic PCA-based partition and several PointNet++-based configurations. The results should therefore be interpreted as empirical evidence regarding method selection and annotation requirements rather than as evidence of a new segmentation architecture.

A key finding is that the relative advantage of rule-based and learning-based methods depends strongly on the supervision setting. When no manually annotated training labels were available, the direct PCA partition was more reliable than training a model entirely on PCA-derived pseudo-labels. The rule-based method therefore provides a reproducible initial baseline for organizing poultry point clouds or generating coarse anatomical labels when annotation resources are limited. However, the present experiments were conducted on an offline single-view dataset and do not demonstrate deployment performance in commercial poultry environments.

This result can be further explained by the supervision mechanism itself. Under pseudo-label supervision, the learning-based models were trained entirely on labels generated by the PCA Rule. As a result, the supervision signal inherited the systematic geometric simplifications and labeling bias of the rule-based partition. Without additional human-corrected annotations, the PointNet++ models were effectively learning from a noisy teacher whose decision boundary was already constrained by the assumptions of the PCA-based split. Consequently, the student models could not reliably exceed the upper bound imposed by the pseudo-label quality, which explains why the PCA Rule remained superior under purely pseudo-label supervision.

Human annotations changed this relationship by providing information beyond the fixed geometric partition. The learning-based methods could then model anatomical and postural variation that constant positional thresholds could not represent. The effects of the structural components also varied with the supervision setting: rule-derived priors were more useful when annotations were limited, whereas Axis-Position information became competitive with increased human supervision. These findings indicate that no single structural enhancement should be regarded as universally optimal; its value depends on the quality and quantity of the available labels.

Nevertheless, the advantage of the PCA Rule under the zero-human-annotation condition should not be interpreted as evidence that reducing annotation is more important than segmentation accuracy. Once human-annotated training data were available, the learning-based methods surpassed the rule-based reference. Therefore, for applications requiring accurate anatomical boundaries or reliable region-specific measurements, human-supervised learning-based segmentation remains preferable. The PCA Rule should be understood primarily as a training-free reference for situations in which manually annotated training data are unavailable.

The additional backbone comparison further indicates that the observed results were not restricted entirely to the original PointNet++ implementation. Under the controlled 1400-record protocol, both PointNeXt-S and PointMLP achieved higher mean mIoU than PointNet++, and PointNeXt-S combined the highest mean mIoU with the lowest parameter count and the smallest variation across seeds. Nevertheless, these results should not be interpreted as a definitive ranking of the three architectures. A common training protocol was used to limit architecture-specific tuning, and only three independent seeds were available. The comparison therefore demonstrates that the evaluation setting can accommodate and differentiate more recent point-cloud architectures, while broader conclusions will require additional models, architecture-specific optimization, and independent datasets.

The limited improvement at the highest annotation level indicates diminishing returns under the current dataset scale, anatomical definition, and model family. Specifically, increasing the annotation budget from 1000 to 1400 samples improved the best mIoU only from 0.7778 to 0.7783, corresponding to an absolute increase of 0.0005. This observation does not establish a universally optimal annotation number, because the saturation point may change with population diversity, annotation consistency, network architecture, and acquisition conditions. It does, however, suggest that after a certain level of within-distribution annotation, improving label quality or expanding sample diversity may be more informative than adding further samples from the same data distribution.

### 5.2. Biological and Practical Implications

From a biological perspective, head–body–tail segmentation provides a coarse but anatomically interpretable representation of poultry body structure. The body region contains most of the trunk surface represented in the top-view point cloud, whereas the head and tail are peripheral regions with different shapes, orientations, and degrees of feather coverage. Separating these regions provides a spatial basis for extracting region-specific geometric descriptors and for examining whether peripheral structures influence measurements intended to characterize trunk size or body conformation. Nevertheless, this three-region partition should be regarded as an intermediate representation rather than as a complete description of poultry anatomy.

From a precision poultry farming perspective, this representation may support research on automated regional measurement, interpretable phenotypic feature extraction, and models that combine global body geometry with local anatomical information. The comparison across annotation budgets also informs method selection under different resource conditions: a geometric rule may be useful for preliminary data organization when manual labels are unavailable, whereas learning-based methods become preferable when reliable annotations can be obtained. Future studies should evaluate these possibilities using more diverse poultry populations, multi-view or temporal point clouds, finer anatomical categories, and reference traits explicitly associated with individual body regions.

The downstream regression experiment served as auxiliary validation of the anatomical representation rather than as an independent methodological contribution. The finding that whole-body geometry remained highly informative is consistent with the nature of total body-weight prediction, which depends on the overall size and conformation of the animal. Nevertheless, the comparison among the fusion strategies provides information beyond a simple whole-body-versus-individual-part comparison. Neither automatically learned part weighting nor a manually specified anatomical ratio produced a consistent advantage, indicating that the contribution of each region cannot be represented adequately by a single fixed scalar weight.

Independently encoding the head, body, and tail also reduced performance relative to the Whole-body PointNet++ model, suggesting that anatomical separation removed useful cross-region geometric relationships. The Hybrid Residual model retained the whole-body representation and used part-aware features as a correction term, producing results close to, but not better than, the whole-body model. These findings do not establish part-aware regression as a superior method. Instead, they show that anatomical decomposition is better treated as supplementary information under the current conditions and identify a limitation that should be considered when segmentation outputs are used in downstream phenotyping.

The regression inputs in this experiment were obtained from human-annotated anatomical regions rather than from PCA Rule or PointNet++ predictions. Consequently, the present results do not demonstrate that PointNet++-generated segmentation improves weight prediction relative to PCA-generated segmentation. Instead, they isolate the effect of anatomical decomposition under a reference segmentation condition. Accurate intermediate segmentation is expected to be important in an operational pipeline, but its quantitative effect on downstream prediction can only be established through an end-to-end comparison using automatically generated regions.

### 5.3. Limitations and Future Work

This study has several limitations that restrict the generalizability of its findings. First, all samples were derived from a previously collected single-view dataset acquired from a top-down perspective. These point clouds primarily represent the dorsal and upper-body surfaces of individual chickens and do not capture the complete three-dimensional anatomy. Occluded regions and structures located below or beside the visible surface are therefore underrepresented. The conclusions cannot be assumed to apply directly to multi-view systems, freely moving birds, crowded poultry houses, or acquisition devices with different geometric and noise characteristics. Second, the annotation scheme divides each chicken into only three coarse regions. Although this partition provides an interpretable representation for the present comparison, it does not distinguish finer structures such as the neck, wings, legs, or subdivisions within the trunk and tail. Boundary definitions may also be affected by posture, feather distribution, and individual morphological variation. Third, although the architecture comparison included PointNet++, PointNeXt-S, and PointMLP, it covered only a limited subset of available point-cloud segmentation approaches. Moreover, the architectures were evaluated using a common training protocol without architecture-specific hyperparameter optimization. The observed relationships among architecture, supervision type, and annotation budget may therefore differ for transformer-based, graph-based, sparse-convolution, or other point-cloud models. Accordingly, the findings should be interpreted as relative trends under the current dataset, anatomical definition, and experimental protocol rather than as universal performance limits for poultry point-cloud segmentation. Inference latency was not benchmarked in the present study. The PCA Rule and PointNet++ were implemented using different computational procedures and may operate on different devices, making a meaningful runtime comparison dependent on hardware, software optimization, input size, and deployment configuration. Accordingly, this study does not draw conclusions regarding their relative inference speed.

Finally, the downstream regression experiment used human-annotated anatomical regions and therefore evaluated the information value of anatomical decomposition under a reference segmentation condition. It did not quantify how errors from PCA-based or learning-based automatic segmentation propagate into weight prediction. An end-to-end comparison using automatically generated regions is required before the practical effect of segmentation accuracy on downstream phenotyping can be established.

Future research should evaluate the task on independent datasets containing greater variation in breed, sex, age, body size, posture, and production environment. Multi-view and temporal point clouds should be investigated to reduce the incomplete anatomical representation caused by single-view acquisition. The annotation scheme could also be extended to finer anatomical categories when these categories are sufficiently visible and can be labeled consistently. Comparisons with a broader range of architectures, including transformer-based, graph-based, and sparse-convolution models, together with validation across different acquisition devices, would further clarify the generalizability of the observed performance trends. For downstream phenotyping, region-specific reference measurements and external-test populations would be required to determine whether anatomically decomposed features provide benefits for traits other than total body weight.

## 6. Conclusions

This study constructed a segmentation-oriented evaluation setting for head–body–tail segmentation of poultry 3D point clouds and compared a PCA-based geometric rule with several PointNet++-based methods under pseudo-label supervision and three manual-annotation budgets. By supplementing a previously published single-view point-cloud dataset with point-wise anatomical annotations and fixed experimental partitions, the study provides a controlled basis for comparing rule-based and learning-based segmentation methods.

Under pseudo-label supervision, the PCA Rule outperformed the models trained entirely on PCA-derived labels. When human annotations were introduced, the learning-based methods surpassed the rule-based baseline. The limited improvement from the 1000-shot to the 1400-shot setting suggests diminishing returns under the current dataset and model family, but should not be interpreted as a universal annotation threshold.

The auxiliary weight-regression experiment showed that anatomical decomposition did not improve upon the complete whole-body representation under the present conditions. Its role in this study was therefore to evaluate the downstream consequences and limitations of part-aware representation rather than to introduce a new weight-prediction method.

Overall, the findings indicate that the relative performance of geometric and learning-based segmentation methods depends on both the source and quantity of supervision. These conclusions are limited to the present single-view dataset, coarse three-class annotation scheme, and PointNet++-based model family. Validation using independent poultry populations, additional acquisition conditions, and other point-cloud architectures is required before the findings can be generalized more broadly.

## Figures and Tables

**Table 1:** Experimental settings under different supervision regimes and annotation budgets.

Setting	Training Labels	Validation Labels	Common Test Labels	Notes
Pseudo-label supervision	Full pseudo-labeled set	Internal split	Same fixed 500-sample human test set	No human labels used for training
500-shot full supervision	400 human-annotated samples	100 human-annotated samples	Same fixed 500-sample human test set	500 human-labeled samples in total
1000-shot full supervision	900 human-annotated samples	100 human-annotated samples	Same fixed 500-sample human test set	1000 human-labeled samples in total
1400-shot full supervision	1300 human-annotated samples	100 human-annotated samples	Same fixed 500-sample human test set	1400 human-labeled samples in total

**Table 2:** Experimental configurations of the downstream weight regression models.

Method	Input Representation	Fusion Strategy	Backbone	Loss
Lightweight Whole-body Baseline	whole-body point cloud	direct regression	lightweight point encoder	Smooth L1/regression loss
Softmax Part-aware	head, body, tail point clouds	softmax-weighted feature fusion	lightweight point encoder	Smooth L1/regression loss
Fixed-weight Part-aware	head, body, tail point clouds	fixed-weight fusion	lightweight point encoder	Smooth L1/regression loss
Pure Concat Part-aware	head, body, tail point clouds	feature concatenation	lightweight point encoder	Smooth L1/regression loss
Whole-body PointNet++	whole-body point cloud	direct regression	PointNet++	Huber
Pure Part-aware PointNet++	head, body, tail point clouds	feature concatenation	PointNet++	Huber
Hybrid Residual PointNet++	whole body + head, body, tail point clouds	whole-body main branch + part-aware residual correction	PointNet++	Huber

**Table 3:** Segmentation performance of the training-free PCA Rule and PointNet++-based models under different training-supervision settings.

Training Supervision	Method	mIoU	Head IoU	Body IoU	Tail IoU	Accuracy
Pseudo-label	Baseline	0.5979	0.5523	0.8648	0.3766	0.8614
	Baseline+AP	0.6009	0.5114	0.8796	0.4116	0.8684
	Baseline+AP+OL	0.5705	0.4747	0.8700	0.3667	0.8536
	Baseline+RP	0.5936	0.5060	0.8761	0.3988	0.8647
	Baseline+BS	0.5935	0.5036	0.8784	0.3984	0.8669
	Baseline+RP+BS	0.6165	0.5449	0.8761	0.4286	0.8718
500 shot	Baseline	0.6443	0.6489	0.9146	0.3693	0.9053
	Baseline+AP	0.7107	0.6987	0.9235	0.5099	0.9221
	Baseline+AP+OL	0.6893	0.6788	0.9200	0.4692	0.9173
	Baseline+RP	0.7250	0.7188	0.9227	0.5333	0.9247
	Baseline+BS	0.6749	0.6785	0.9177	0.4286	0.9146
	Baseline+RP+BS	0.6948	0.6678	0.9215	0.4951	0.9181
1000 shot	Baseline	0.7501	0.7586	0.9324	0.5592	0.9330
	Baseline+AP	0.7778	0.7905	0.9306	0.6123	0.9375
	Baseline+AP+OL	0.7669	0.7750	0.9302	0.5956	0.9357
	Baseline+RP	0.7651	0.7860	0.9288	0.5806	0.9349
	Baseline+BS	0.7577	0.7782	0.9303	0.5646	0.9349
	Baseline+RP+BS	0.7483	0.7611	0.9296	0.5542	0.9322
1400 shot	Baseline	0.7653	0.7834	0.9284	0.5840	0.9350
	Baseline+AP	0.7716	0.8081	0.9191	0.5877	0.9302
	Baseline+AP+OL	0.7783	0.8000	0.9267	0.6080	0.9357
	Baseline+RP	0.7662	0.7815	0.9233	0.5937	0.9322
	Baseline+BS	0.7638	0.7864	0.9275	0.5774	0.9347
	Baseline+RP+BS	0.7645	0.7672	0.9240	0.6023	0.9325
None (training-free)	PCA Rule	0.6874	0.6530	0.8761	0.5330	0.8920

Abbreviations: PCA, principal component analysis; Baseline, the basic PointNet++ segmentation model using only xyz coordinates as input; AP, Axis Position; OL, Order Loss; RP, Rule Prior; BS, Boundary Supervision; RP+BS, the combination of Rule Prior and Boundary Supervision. Note: The PCA Rule is reported only once because it does not require model training and produces the same predictions regardless of the human-annotation budget. Pseudo-label supervision refers to PointNet++-based models trained with point-wise labels generated automatically by the PCA Rule; it is not an unsupervised-learning condition.

**Table 4:** Controlled comparison of representative point-cloud segmentation architectures under the 1400-record supervision setting.

Method	mIoU	Head IoU	Body IoU	Tail IoU	Accuracy	Parameters
PointNet++	0.7170 ± 0.0230	0.6862	0.9224	0.5425	0.9232	1.821 M
PointNeXt-S	0.7656 ± 0.0035	0.7962	0.9177	0.5830	0.9279	0.977 M
PointMLP	0.7585 ± 0.0150	0.7795	0.9214	0.5747	0.9290	16.744 M

Note: mIoU is reported as mean ± standard deviation across three independent training seeds. Class-wise IoU and accuracy are means across seeds. All architectures were evaluated using the same development and external-test partitions and the same general training protocol.

**Table 5:** Computational capacity comparison of the PCA Rule and learning-based segmentation methods under the 3072-point input setting.

Method	Device	Params (M)	FP32 Size (MB)	FLOPs (G)	Median Time (ms)	CUDA Memory (MB)
PCA Rule	CPU	0.000	0.00	0.00	0.76	-
Baseline	GPU	1.821	6.95	8.55	905.78	116.34
Baseline+AP	GPU	1.822	6.95	8.56	817.26	116.35
Baseline+AP+OL	GPU	1.822	6.95	8.56	817.95	116.35
Baseline+RP	GPU	1.823	6.95	8.60	834.04	116.40
Baseline+BS	GPU	1.822	6.95	8.55	836.76	116.37
Baseline+RP+BS	GPU	1.823	6.95	8.61	505.80	116.43

**Table 6:** Performance comparison of different downstream weight regression strategies.

Method	MAE (g)	MAPE (%)	Pearson r
Lightweight Whole-body Baseline	207.85	15.36	0.3651
Softmax Part-aware	215.31	15.66	0.2275
Fixed-weight Part-aware	207.51	15.12	0.3571
Pure Concat Part-aware	203.48	14.91	0.4016
Whole-body PointNet++	138.56	10.06	0.7316
Pure Part-aware PointNet++	213.43	15.51	0.3390
Hybrid Residual PointNet++	141.95	10.31	0.7291

**Table 7:** Downstream weight-estimation performance of the frozen Hybrid Residual PointNet++ model using manually annotated and PCA-generated anatomical parts.

Anatomical Part Source	MAE (g)	MAPE (%)	RMSE (g)
Manual annotation	142.79	10.41	192.75
PCA Rule	143.86	10.50	192.78
Anatomical part source	MAE (g)	MAPE (%)	RMSE (g)

Note: The comparison was conducted on the same 393-record test subset using a frozen Hybrid Residual PointNet++ model. The whole-body branch, trained model parameters, and evaluation protocol were held fixed; only the head, body, and tail inputs to the local branches were replaced. The mean paired absolute-error difference for PCA-generated versus manually annotated parts was 1.07 g (95% CI, −2.03 to 4.17 g; Holm-adjusted *p* = 0.821).

## Data Availability

The point-cloud data used in this study were derived from the dataset described in reference [14]. The data and the point-wise head, body, and tail annotations generated for the present study are available from the corresponding author upon reasonable request.
